# Development of Rapid Enzyme-Linked Immunosorbent Assays for Detection of Antibodies to Burkholderia pseudomallei

**DOI:** 10.1128/JCM.02856-15

**Published:** 2016-04-25

**Authors:** Vichaya Suttisunhakul, Vanaporn Wuthiekanun, Paul J. Brett, Srisin Khusmith, Nicholas P. J. Day, Mary N. Burtnick, Direk Limmathurotsakul, Narisara Chantratita

**Affiliations:** a Department of Microbiology and Immunology, Faculty of Tropical Medicine, Mahidol University, Bangkok, Thailand; b Mahidol-Oxford Tropical Medicine Research Unit, Faculty of Tropical Medicine, Mahidol University, Bangkok, Thailand; c Department of Microbiology and Immunology, University of South Alabama, Mobile, Alabama, USA; d Center for Tropical Medicine & Global Health, Nuffield Department of Medicine, University of Oxford, Oxford, United Kingdom; e Department of Tropical Hygiene, Faculty of Tropical Medicine, Mahidol University, Bangkok, Thailand

## Abstract

Burkholderia pseudomallei, the causative agent of melioidosis, is an environmental bacillus found in northeast Thailand. The mortality rate of melioidosis is ∼40%. An indirect hemagglutination assay (IHA) is used as a reference serodiagnostic test; however, it has low specificity in areas where the background seropositivity of healthy people is high. To improve assay specificity and reduce the time for diagnosis, four rapid enzyme-linked immunosorbent assays (ELISAs) were developed using two purified polysaccharide antigens (O-polysaccharide [OPS] and 6-deoxyheptan capsular polysaccharide [CPS]) and two crude antigens (whole-cell [WC] antigen and culture filtrate [CF] antigen) of B. pseudomallei. The ELISAs were evaluated using serum samples from 141 culture-confirmed melioidosis patients from Thailand along with 188 healthy donors from Thailand and 90 healthy donors from the United States as controls. The areas under receiver operator characteristic curves (AUROCC) using Thai controls were high for the OPS-ELISA (0.91), CF-ELISA (0.91), and WC-ELISA (0.90), while those of CPS-ELISA (0.84) and IHA (0.72) were lower. AUROCC values using U.S. controls were comparable to those of the Thai controls for all ELISAs except IHA (0.93). Using a cutoff optical density (OD) of 0.87, the OPS-ELISA had a sensitivity of 71.6% and a specificity of 95.7% for Thai controls; for U.S. controls, specificity was 96.7%. An additional 120 serum samples from tuberculosis, scrub typhus, or leptospirosis patients were evaluated in all ELISAs and resulted in comparable or higher specificities than using Thai healthy donors. Our findings suggest that antigen-specific ELISAs, particularly the OPS-ELISA, may be useful for serodiagnosis of melioidosis in areas where it is endemic and nonendemic.

## INTRODUCTION

Melioidosis is a severe illness caused by Burkholderia pseudomallei, a Gram-negative bacterium that is naturally found in moist soils and surface waters in tropical and subtropical regions. Human infections typically result from contact with contaminated environmental sources by inoculation, ingestion, or inhalation. B. pseudomallei is an important cause of community-acquired infections in areas where melioidosis is highly endemic, such as Southeast Asia and northern Australia. Mounting evidence in the literature suggests that B. pseudomallei exists in the environment on several continents (1) and that cases of melioidosis may be going unrecognized due to a lack of good laboratory diagnostic tools and epidemiological data (2, 3). The number of case reports of melioidosis from various countries is continually increasing in the literature, as awareness of the disease is improving worldwide. Several recent cases of travel-associated melioidosis have also been documented in individuals returning to regions where the disease is nonendemic from Southeast Asia, Africa, and the Caribbean (4–12).

The clinical spectrum of melioidosis is broad, and symptoms often mimic other illnesses, which can make diagnosis challenging. Disease presentations range from skin and soft tissue abscesses to acute pneumonia and septicemia with fatal outcomes. In northeast Thailand, the mortality rate is ∼40% but can reach as high at 90% in severe sepsis (13), which can occur within 24 to 48 h of the onset of symptoms (14). To improve patient outcomes, rapid diagnosis of melioidosis is critical so that appropriate antibiotic therapy can be administered without delay. At present, isolation of B. pseudomallei from clinical specimens followed by biochemical identification is routinely used in hospital laboratories. Although this technique is specific and relatively low cost, definitive identification of B. pseudomallei requires experience and can be time-consuming (2 to 7 days). In addition, culture is not a perfect gold standard because it has only 60% sensitivity (15). Possible explanations for this may be the low B. pseudomallei numbers in clinical samples (16) or the presence of unculturable forms of the organism that have been associated with previous antibiotic treatment in some patients.

To achieve early diagnosis of melioidosis, detection of B. pseudomallei in clinical specimens by other techniques has been developed. For example, immunological tests such as immunofluorescent assays (IFAs) and molecular methods such as real-time PCR and loop-mediated amplification (LAMP) have been used. These methods have been evaluated in our prospective studies in Sappasithiprasong Hospital, northeast Thailand. While these tests are highly specific (>95%), the sensitivities are only 48% for IFA (17), 34% to 61% for PCR based on *TTS1* and 16S rRNA genes, respectively, and 44% for LAMP based on the *TTS1* gene (18, 19). Attempts to detect B. pseudomallei directly in blood by IFA or latex agglutination were unsuccessful; however, these methods were useful if the bacteria in hemoculture were enriched by incubation for 1 or 2 days (20, 21). A lateral flow immunoassay (LFI) for the detection of the B. pseudomallei 6-deoxyheptan capsular polysaccharide (CPS) antigen has been developed and shown to be highly specific but had low sensitivity (40%) when used with whole-blood specimens (22, 23).

Serology is commonly used to test for evidence of exposure to B. pseudomallei. In general, serological assays are easy to perform and inexpensive and thus can reduce the time for laboratory diagnosis. A melioidosis-specific indirect hemagglutination assay (IHA) is routinely used but has potential problems, including a short shelf-life and unstandardized antigen preparations. The diagnostic sensitivity of IHA is low (56% to 70%), and the specificity is variable depending on the background seropositivity of healthy individuals in the population being tested (3, 24). We have recently developed two rapid latex agglutination assays based on B. pseudomallei surface polysaccharides, the O-polysaccharide (OPS) component of lipopolysaccharide (LPS) or CPS, and demonstrated that OPS is a promising antigen for serodiagnosis of melioidosis in areas where the disease is nonendemic (24). The OPS-latex agglutination test had 84.4% sensitivity and 98% specificity with U.S. donor serum samples but only 57% specificity with Thai donor serum samples (24).

Several factors can contribute to the poor performance of antibody detection by agglutination methods. False-positive agglutination may occur as a result of using high serum concentrations or may be due the presence of background antibody titers in individuals who have been exposed to B. pseudomallei antigens or to antigenically related environmental organisms (3). False-negatives may result from a prozone phenomenon due to the use of an inappropriate serum dilution (24) or to the use of poorly defined antigens. Development of assays with increased sensitivity for the detection of antigen-antibody reactions and reduced nonspecific interactions would have more potential for use in areas where melioidosis is endemic. Among these, enzyme-linked immunosorbent assay (ELISA) is a standard quantitative method that may be a platform for the development of improved rapid point-of-care tests. ELISAs have been previously evaluated in a clinical setting in Thailand, but in their current format are suboptimal since they are time-consuming (>24 h) and lack the performance for clinical use (25, 26).

To further improve the specificity and enhance speed of antibody detection, we have developed four rapid ELISAs based on two purified polysaccharide antigens (OPS and CPS) and two crude antigens of B. pseudomallei. These assays were evaluated using serum samples from culture-proven melioidosis patients, other bacterial infection patients in Thailand, and healthy donors from northeast Thailand and the United States. The performance of these ELISAs in this study was compared with the standard IHA.

## MATERIALS AND METHODS

### Serum samples.

Initially, three sets of human serum samples were used anonymously to evaluate the assays as described previously (24). These included 141 on-admission serum samples from culture-confirmed B. pseudomallei-infected patients who were admitted to Sappasithiprasong Hospital in Ubon Ratchathani, northeast Thailand (27), 188 serum samples obtained from healthy donors from the same area in northeast Thailand, and 90 serum samples obtained from healthy U.S. donors (Innovative Research, Novi, MI, USA). Another three sets of on-admission anonymous human serum samples were used to further evaluate the specificity of the ELISAs. These included (i) 20 acid-fast-stain-positive tuberculosis patients from Chiangrai, north Thailand, (ii) 50 culture-proven scrub typhus patients from Udon Thani, northeast Thailand, and (iii) 50 culture-proven leptospirosis patients from Udon Thani, northeast Thailand. All serum samples were stored at −80°C. The study was approved by the Ethics Committee of Faculty of Tropical Medicine, Mahidol University (approval number MUTM 2014-079-02).

### Preparation of antigens.

B. pseudomallei LPS (type A) and CPS were extracted from the select-agent-excluded strains RR2808 (CPS mutant) (28) or RR2683 (OPS mutant) (29), respectively, using a modified hot phenol procedure (30). Purified OPS and CPS antigens were then obtained via acid hydrolysis and gel permeation chromatography as previously described (29). No protein or nucleic acid contamination was detected in the preparations as estimated by bicinchoninic acid (BCA) assay (Pierce) and *A*_260/280_ measurements, respectively. Whole-cell (WC) antigen was extracted from B. pseudomallei K96243 (from a Thai patient in northeast Thailand; expresses type A OPS) as described previously (25). Briefly, the bacteria were grown on Trypticase soy agar at 37°C for 2 days, harvested into 30 ml of phosphate-buffered saline (PBS) pH 7.2, and killed at 80°C for 1 h. The preparation was centrifuged at 10,000 × *g* for 10 min, and the supernatant was used as the antigen. Culture filtrate (CF) antigen was prepared from pool antigens of two clinical B. pseudomallei isolates from northeast Thailand, strains 199a and 207a, as described previously for IHA antigen preparation (31, 32). Briefly, the bacteria were cultured in rice broth medium (33) at 37°C for 14 days. The culture was autoclaved at 121°C for 15 min followed by centrifugation. The supernatant was sterilized by filtration using a 0.2-μm filter membrane.

### ELISA.

The rapid ELISA was developed using two purified polysaccharide antigens and two crude antigens of B. pseudomallei as follows: (i) OPS, (ii) CPS, (iii) whole-cell (WC) antigen, and (iv) culture filtrate (CF) antigen. OPS and CPS were the same antigens used for our latex agglutination assays (24), and WC antigen was heat-killed B. pseudomallei antigen (25). CF was the antigen used for sensitized sheep red blood cells in IHA as a standard protocol at the Mahidol Oxford Tropical Medicine Research Unit as described above and was used for comparison between ELISA and IHA.

The optimal concentrations of antigens were determined by using pooled melioidosis and pooled healthy sera (5 patients with culture-confirmed melioidosis and 5 healthy Thai donors who were IHA negative). The antigens were diluted in 0.05 M carbonate buffer, pH 9.6, at a dilution range of 0 to 3 μg/ml (2-fold serial dilution starting from 3 μg/ml). The optimal concentration of serum dilution was defined by using 3 sets of human serum samples, including culture-confirmed melioidosis (*n* = 20), healthy Thai donors (*n* = 20), and healthy U.S. donors (*n* = 20) at a dilution range of 1:1,000 to 1:128,000 (2-fold serial dilution). Each antigen was added in duplicate to a 96-well ELISA plate (Nunc MaxiSorp U-bottom 96-Well plates; Thermo Scientific, Denmark) in a fixed volume of 50 μl per well and was incubated overnight in a moist chamber at 4°C. The plates were then washed 4 times with 300 μl of wash buffer (0.05% Tween 20 in PBS) using a HydroFlex microplate washer (Tecan, Switzerland) followed by blocking at 37°C for 2 h with 200 μl of 5% skim milk in PBS and were further washed as described above. Fifty microliters of serum diluted in PBS containing 1% bovine serum albumin and 0.05% Tween 20 was added to wells in duplicate and incubated at room temperature for 30 min. After washing, 50 μl of 1:2,000 dilution of horseradish peroxidase-conjugated rabbit antihuman IgG (Dako, Copenhagen, Denmark) was added, and the mixture was incubated at room temperature for 30 min. After washing 4 times, enzymatic activity was determined using 50 μl of TMB substrate solution (Invitrogen, Camarillo, CA, USA) incubated at room temperature for 15 min. The reaction was stopped with 50 μl of 1 N HCl, and the absorbance value (optical density [OD]) was determined at a wavelength of 450 nm using a microtiter plate reader (Promega, Madison, WI, USA).

Following optimization for antigen concentration and serum dilution, the plates were prepared for ELISA using the optimized antigen concentration. After washing and blocking, the plates were dried at room temperature for 2 h and were kept in a sealed plastic bag at 4°C until use. On the day that the ELISA was performed, the plate was removed from 4°C and was left at room temperature for 15 min. Based on the results of the optimization experiments, the antibody was determined in individual serum samples in duplicate for all ELISAs using the conditions described above.

### Indirect hemagglutination assay.

The antigen for IHA was prepared from culture filtrate of B. pseudomallei (CF antigen) as described above. The antigens were pooled and used to sensitize sheep red blood cells for testing agglutinations with serial dilution of inactivated serum samples in 96-well plates as previously described (24, 32). The results were read at the highest antibody titer that showed agglutination after incubating at room temperature for 2 h. Positive IHA results were determined at a cutoff dilution of ≥1:160 (24, 32).

### Determination of antibody to B. pseudomallei wild type and OPS mutant.

To examine the proportion of antibodies specific to OPS in comparison to antibodies against other B. pseudomallei antigens, WC antigens were prepared from the wild-type strain K96243 and an OPS mutant (Δ*wbiD* K96243) and were used to conduct ELISAs as described above. The OPS mutant defective in *wbiD* (BPSL2677) was constructed as described in our previous study (34). The ELISAs were performed with serum from 5 randomly selected melioidosis patients at a dilution range of 1:1,000 to 1:128,000 (2-fold serial dilution).

### Statistical analysis.

Statistical analyses were performed using Stata version 12 (StataCorp LP, College Station, TX, USA). A receiver operator characteristic (ROC) curve was constructed to illustrate the impact of shifting the positive cutoff value on true-positive (sensitivity) and false-positive (1 − specificity) rates. Areas under the ROC curves (AUROCC) were compared using a nonparametric approach as described by DeLong et al. (35). The Mann-Whitney test was used to test the difference of median between different serum groups. Spearman's rank correlation was performed to determine the correlation coefficient for all pairs of the tests (36, 37). Differences were considered statistically significant if the *P* value was <0.05. The ELISA data of the melioidosis group and Thai donors were evaluated separately from the data of the melioidosis group and U.S. donors using three different OD cutoff values at specificities of 85%, 90%, and 95%.

## RESULTS

### Optimization of rapid ELISAs.

The optimal conditions for ELISAs were initially determined using pooled serum from either melioidosis patients or healthy donors. The optimized concentrations of the coating antigens were determined to be 1 μg/ml for OPS, 1 μg/ml for CPS, 250 ng/ml for WC, and 1.5 μg/ml for CF. The ELISAs were standardized by precoating plates with antigen overnight, blocking, and then storing dried at 4°C until use. To determine the optimal serum dilution for screening the serum samples, we tested 2-fold serial dilutions from culture-confirmed melioidosis patients, Thai donors, and U.S. donors. The greatest differences in OD values at 450 nm between the melioidosis group and healthy donors were obtained at serum dilution of 1:2,000 with OPS, WC, and CF antigens and 1:1,000 with CPS antigen. The primary antibody incubation was therefore performed using a serum dilution of 1:2,000 for the OPS-, WC-, and CF-ELISA and of 1:1,000 for CPS-ELISA at room temperature (25°C) for 30 min. The assay was standardized throughout the study using these dilutions for all serum samples. The total time for the ELISAs following the addition of the serum samples to the precoated/blocked plates was 1.5 h.

### Detection of specific antibodies in serum samples by ELISA and IHA.

**FIG 1:**
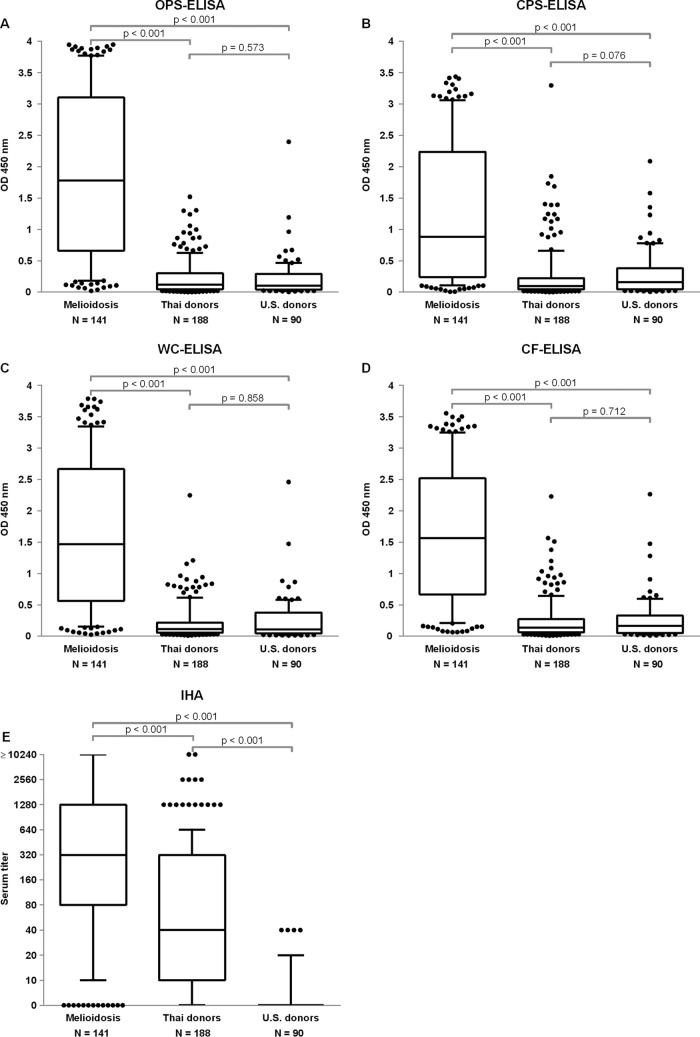
Results of ELISA of four different antigens and IHA using serum samples from melioidosis patients, Thai healthy donors, and U.S. healthy donors. Box plots represent 25th and 75th percentile boundaries in the box with the median line within the box; the whiskers indicate the 10th and 90th percentiles. The plots show OD at 450 nm for each antigen: (A) OPS, (B) CPS, (C) WC, (D) CF antigens, and (E) IHA serum titer.

A total of 419 serum samples were initially used to evaluate the four ELISAs and were compared with the results of IHA. These include on-admission serum samples from culture-proven meliodosis patients (*n* = 141), Thai healthy donors (*n* = 188), and U.S. healthy donors (*n* = 90) (24). Quantitative results of OD values in all ELISAs and IHA titers are summarized in Fig. 1. In all ELISAs, the median OD value of the melioidosis group was statistically different from Thai donors and U.S. donors (*P* < 0.001 for all ELISAs). The median OD of Thai donors was not different from that of U.S. donors. In contrast to the ELISAs, the IHA titers of Thai donors were statistically higher than those of U.S donors (median titers, 1:40 [interquartile range, IQR, 10 to 320] versus 0; *P* < 0.001).

### ROCs of ELISAs and IHA.

**FIG 2:**
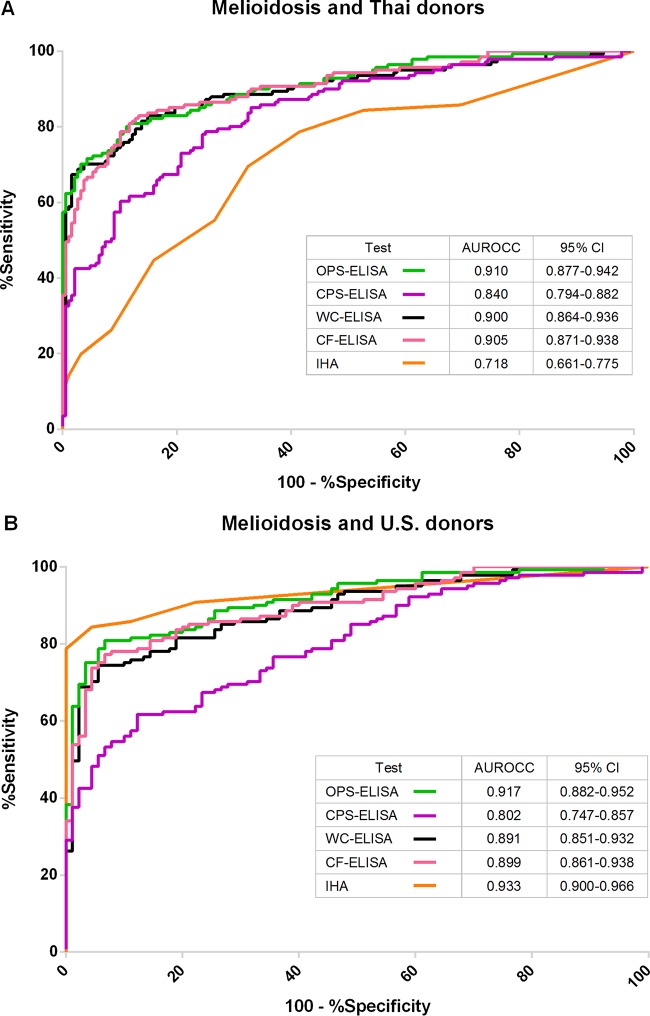
Receiver operating characteristics (ROC) plots of the ELISA and IHA results with different groups of serum. Serum from melioidosis patients versus Thai healthy donors (A) and Serum form melioidosis patients versus U.S. healthy donors (B). The serum samples were diluted 1:2,000 for all ELISAs and 1:1,000 for CPS-ELISA and were used in duplicated on the plates coated with OPS, CPS, WC, and CF antigens.

ROC was plotted by calculating the sensitivity and specificity of increasing numbers of the true-positive rate and false-positive rate (1 − specificity). The results for comparisons of all ELISAs and IHA using the melioidosis group and Thai donors are shown in Fig. 2A, and those using the melioidosis group and U.S. donors are shown in Fig. 2B. When the results of Thai donors were analyzed, the areas under the receiver operator characteristic curves (AUROCCs) for predicting melioidosis were highest and comparable between 0.90 and 0.91 for the OPS-ELISA, CF-ELISA, and WC-ELISA (Fig. 2A). The AUROCC of the CPS-ELISA was 0.84, which was significantly lower than those of the other three ELISAs (CPS-ELISA versus OPS-ELISA, *P* = 0.002; CPS-ELISA versus WC-ELISA, *P* < 0.001; CPS-ELISA versus CF-ELISA, *P* < 0.001). The AUROCC of the IHA was 0.72, which was the lowest among all assays (IHA versus OPS-ELISA, IHA versus CPS-ELISA, IHA versus WC-ELISA, IHA versus CF-ELISA; *P* < 0.001 for all comparisons). When the results from the U.S. donors were analyzed (Fig. 2B), the AUROCCs for predicting melioidosis were high for the OPS-ELISA (0.92), CF-ELISA (0.90), WC-ELISA (0.89), and IHA (0.93). AUROCC of the CPS-ELISA was 0.80, which was the lowest in all assays (CPS-ELISA versus OPS-ELISA, CPS-ELISA versus WC-ELISA, CPS-ELISA versus CF-ELISA, CPS-ELISA versus IHA; *P* < 0.001 for all comparisons).

### Sensitivity and specificity of ELISA and IHA.

We initially analyzed the performance of all four ELISAs in comparison to IHA. A total of 419 serum samples were tested, including 141 on-admission serum samples from melioidosis patients, 188 serum samples from Thai healthy donors from the same province in northeast Thailand, and 90 healthy donors from the United States. The data from the melioidosis group using Thai donors as controls were assessed separately from the data of the melioidosis group using U.S. donors as controls. We estimated three different OD cutoff values at specificities of 85%, 90%, and 95%. The sensitivity and specificity of these assays for the melioidosis group with Thai donors as controls are presented in Table 1. The results demonstrated the greatest and most comparable performances for the OPS-ELISA, CF-ELISA, and WC-ELISA. At a positive cutoff OD of 0.44, the sensitivity and specificity of the OPS-ELISA were 81.6% and 85.1%, respectively. At an increased positive cutoff OD of 0.63, the sensitivity and specificity of the OPS-ELISA were 76.6% and 90.4%, respectively. At a further increased positive cutoff OD of 0.87, the sensitivity and specificity of the OPS-ELISA were 71.6% and 95.7%, respectively. The performance indices were lower with the CPS-ELISA. At a positive cutoff OD of 0.51, the sensitivity and specificity of the CPS-ELISA were 62.4% and 85.6%, respectively. Among the tests evaluated using Thai healthy donors, the performance indices were the lowest with IHA. At a cutoff titer of 1:160, which is routinely used in diagnostic laboratories, the IHA showed 69.5% sensitivity and 67.6% specificity (24). The sensitivity was <45% (at 1:640, 1:1,280, and 1:2,560) when the different cutoff titers were used to obtain specificities of 84.0%, 91.5%, and 96.8%.

**TABLE 1:** Sensitivity and specificity of the four ELISAs and IHA^*a*^

Assay	Cutoff	% Sensitivity	% Specificity			
		Thai melioidosis patients (*n* = 141)	Thai healthy donors (*n* = 188)	Tuberculosis patients (*n* = 20)	Scrub typhus patients (*n* = 50)	Leptospirosis patients (*n* = 50)
OPS-ELISA	0.44	81.6	85.1	100	88.0	94.0
	0.63	76.6	90.4	100	90.0	98.0
	0.87	71.6	95.7	100	94.0	98.0
CPS-ELISA	0.51	62.4	85.6	100	88.0	92.0
	0.68	57.5	90.9	100	94.0	98.0
	1.18	43.5	95.2	100	98.0	100
WC-ELISA	0.36	83.0	85.1	100	94.0	98.0
	0.61	74.5	90.4	100	96.0	98.0
	0.83	70.2	95.7	100	98.0	100
CF-ELISA	0.46	83.0	85.6	100	92.0	96.0
	0.68	75.2	91.0	100	96.0	98.0
	0.97	66.7	95.7	100	96.0	100
IHA	1:640	44.7	84.0	ND^*b*^	ND	ND
	1:1,280	26.2	91.5	ND	ND	ND
	1:2,560	19.9	96.8	ND	ND	ND

a The assay values were calculated from Thai patients who had melioidosis, tuberculosis, scrub typhus, and leptospirosis as well as Thai healthy donors.

b ND, not done.

The ELISAs were also evaluated using U.S. donors as controls. As shown in Table 2, the OPS-ELISA demonstrated the greatest performance followed by CF-ELISA and WC-ELISA. At a positive cutoff OD of 0.41, the sensitivity and specificity of the OPS-ELISA were 82.3% and 85.6%, respectively. At an increased positive cutoff OD of 0.46, the sensitivity and specificity of the OPS-ELISA were 80.9% and 90.0%, respectively. At a further increased positive cutoff OD of 0.66, the sensitivity and specificity of the OPS-ELISA were 75.2% and 95.6%, respectively. At a positive cutoff OD of 0.87, the sensitivity and specificity of the OPS-ELISA were 71.6% and 96.7%, respectively. The performance of CPS-ELISA was the lowest. At a positive cutoff OD of 0.55, the sensitivity and specificity were only 61.7% and 85.6%, respectively. The performance of the IHA was highest when the test was evaluated in U.S. donors. At cutoff titer of 1:40, for example, the sensitivity and specificity were 84.4% and 95.6%, respectively.

**TABLE 2:** Sensitivity and specificity of the four ELISAs and IHA^*a*^

Assay	Cutoff	% Sensitivity for Thai melioidosis patients (*n* = 141)	% Specificity for U.S. healthy donors (*n* = 90)
OPS-ELISA	0.41	82.3	85.6
	0.46	80.9	90.0
	0.66	75.2	95.6
	0.87^*b*^	71.6	96.7
CPS-ELISA	0.55	61.7	85.6
	0.75	56.0	90.0
	0.95	48.2	95.6
	1.18^*b*^	43.3	95.6
WC-ELISA	0.51	78.0	85.6
	0.57	75.2	90.0
	0.81	70.2	95.6
	0.83^*b*^	70.2	95.6
CF-ELISA	0.54	80.9	85.6
	0.61	78.0	91.1
	0.73	73.7	95.6
	0.97^*b*^	66.7	96.7
IHA	1:10	90.8	77.8
	1:20	85.8	88.9
	1:40	84.4	95.6
	1:2,560^*b*^	26.2	100

a The assay values were calculated from Thai melioidosis patients and U.S. healthy donors.

b OD cutoff at 95% specificity when Thai donors were used as controls.

An additional 120 serum samples from patients with other bacterial infections were used to further evaluate the specificity of all ELISAs. These included tuberculosis patients (*n* = 20), scrub typhus patients (*n* = 50), and leptospirosis patients (*n* = 50). The specificities of ELISA for these groups are presented in Table 1. These samples were stored serum obtained from patients in other provinces where the incidence of these infections were reported. The specificity of ELISAs using these serum samples was comparable to or higher than that of using Thai healthy donors (Table 1). At all three cutoff ODs, the specificity using the tuberculosis group was 100%. The specificities of the OPS-ELISA were comparable between the scrub typhus and Thai healthy donor serum samples. The specificity of the OPS-ELISA for the leptospirosis group was 94% to 98%.

### Correlation between results of ELISAs using different antigens and IHA.

Because the AUROCC values from the OPS-, CF-, and WC-ELISAs were comparable (Fig. 2), we determined whether the antibody responses to the two crude antigens (WC and CF) correlated with the responses elicited by OPS or CPS. For all 539 serum samples, we analyzed the pairwise correlations of the OD results for each pair of the four ELISAs. The correlation of the OD results of the ELISAs and IHA titers were analyzed for 419 serum samples for which IHA data were available. These included serum samples from melioidosis patients, Thai healthy donors, and U.S. healthy donors. As shown in Table 3, the results indicate a strong relatedness between antibody responses to the OPS, WC, and CF antigens (correlation coefficient, rho = 0.9). The CPS-ELISA was less correlated with the OPS-ELISA (rho = 0.7), WC-ELISA (rho = 0.8), and CF-ELISA (rho = 0.8). IHA titers had only moderate correlations with all ELISAs (see Fig. S1 in the supplemental material; correlation coefficients = 0.5 to 0.6).

**TABLE 3:** Correlation between results of ELISA based on four different antigens and IHA^*a*^

Assay	OPS-ELISA	CPS-ELISA	WC-ELISA	CF-ELISA	IHA
OPS-ELISA	1.0000	0.7488	0.9315	0.8957	0.6076
CPS-ELISA		1.0000	0.8301	0.8384	0.5320
WC-ELISA			1.0000	0.9374	0.6045
CF-ELISA				1.000	0.5946
IHA					1.0000

a Correlations between ELISAs were obtained from OD values of all 539 serum samples from melioidosis patients (*n* = 141), Thai healthy donors (*n* = 188), U.S. healthy donors (*n* = 90), tuberculosis patients (*n* = 20), scrub typhus patients (*n* = 50), and leptospirosis patients (*n* = 50). Correlation between ELISAs and IHA were obtained from OD values of ELISA results and IHA titers of 419 serum samples, in which the IHA data were avalilable from melioidosis patients, Thai donors, and U.S. healthy donors. The data presented are pairwise correlation coefficients.

### Comparison of the combined and individual ELISA results.

A summary of the ELISA results for all 539 serum samples used in this study is represented in Fig. S2 in the supplemental material. The combined results for the four ELISAs and the positive results for each individual assay are summarized in Table 4. Comparison between each of the serum groups using a cutoff OD of 95% specificity demonstrated that 50/141 melioidosis patients (35.5%) were positive for all tests and 29/141 patients (20.6%) were negative for all tests. The number of melioidosis serum samples that were positive for a single ELISA was highest for the OPS-ELISA (7/141, 5.0%). The number of melioidosis serum samples positive for any ELISA was 112/141 (79.4%).

**TABLE 4:** Comparison of combination results of four different ELISAs and each ELISA using serum samples from melioidosis patients, Thai healthy donors, U.S. healthy donors, tuberculosis patients, scrub typhus patients, and leptospirosis patients^*a*^

ELISA results	No. of serum samples (%)					
	Melioidosis (*n* = 141)	Thai healthy donors (*n* = 188)	U.S. healthy donors (*n* = 90)	Tuberculosis (*n* = 20)	Scrub typhus (*n* = 50)	Leptospirosis (*n* = 50)
Positive for all ELISAs	50 (35.5)	1 (0.5)	2 (2.2)	0	1 (2.0)	0
Positive for any ELISA	112 (79.4)	17 (9.0)	4 (4.4)	0	4 (8.0)	1 (2.0)
Positive for OPS-ELISA only	7 (5.0)	3 (1.6)	0	0	2 (4.0)	1 (2.0)
Positive for CPS-ELISA only	1 (0.7)	2 (1.1)	0	0	0	0
Positive for WC-ELISA only	0	0	0	0	0	0
Positive for CF-ELISA only	1 (0.7)	1 (0.5)	0	0	1 (2.0)	0
Negative for all ELISAs	29 (20.6)	171 (91.0)	86 (95.6)	20 (100)	46 (92.0)	49 (98.0)

a The cutoff ODs used for OPS-ELISA, CPS-ELISA, WC-ELISA, and CF-ELISA were 0.87, 1.18, 0.83, and 0.97, respectively.

The sensitivity of the combined results of the four ELISAs was higher than that of the OPS-ELISA only (79.4% versus 71.6%; *P* = 0.001), but the specificity was significantly reduced (91.0% versus 95.7%; *P* = 0.004). Moreover, false-positive results of the four ELISAs combined increased compared to those using only the OPS-ELISA (1.6% to 9.0% of Thai healthy donors, 0 to 4.4% of U.S. healthy donors, 4% to 8% of scrub typhus patients) (Table 4).

### Comparison of antibody responses to wild-type B. pseudomallei and an OPS mutant by ELISA.

**FIG 3:**
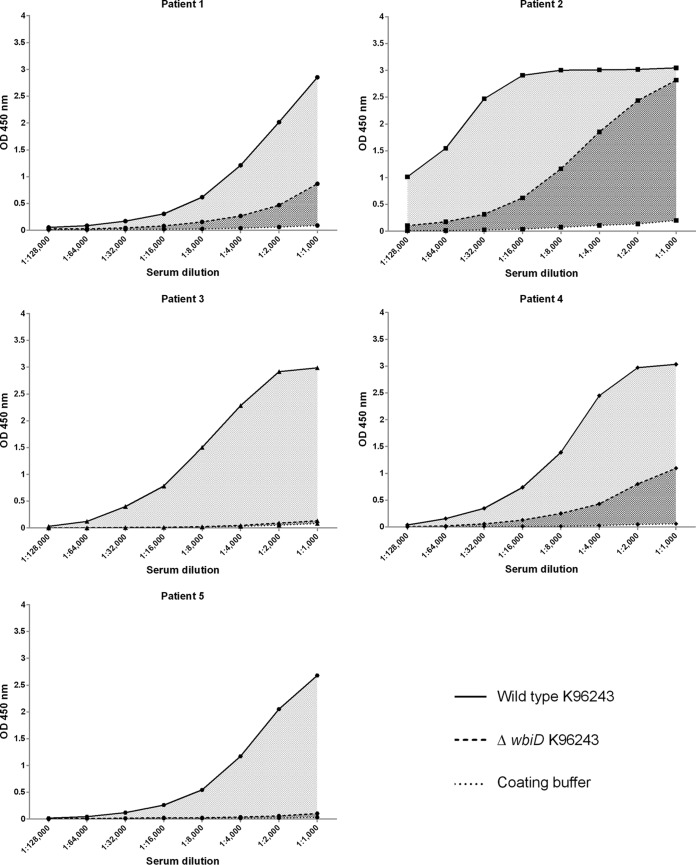
Antibodies to whole-cell antigens of B. pseudomallei K96243 wild type and OPS mutant (Δ*wbiD* K96243). ELISAs were performed in serial dilution using five melioidosis patients.

Since our results showed a strong correlation between the OPS-ELISA and the two ELISAs based on crude antigens (WC-ELISA and CF-ELISA), we next investigated the possibility that OPS might be the predominant antigen recognized by human antibodies in the crude preparations. We determined the antibody titers in serum samples from five individual melioidosis patients by WC-ELISAs using coating antigens prepared from either the wild-type strain (K96243) or an OPS mutant (Δ*wbiD* K96243) (Fig. 3). Our results revealed that the antibody levels in three patients (patients 1, 2, and 4) were markedly decreased in the OPS mutant WC-ELISA compared to those of the wild-type WC-ELISA. Moreover, the antibody titers in the serum samples of two patients (patients 3 and 5) were virtually undetectable when tested against the WC antigen prepared from the OPS mutant.

## DISCUSSION

Since ELISAs can be optimized to have relatively short performance times and are less labor-intensive than some assay formats, they represent an attractive platform for the development of serologic tests. In the present study, our results demonstrated that rapid ELISAs based on highly purified or crude B. pseudomallei antigens are improved methods for serodiagnosis of melioidosis. Compared to the standard IHA, the mean OD values in the ELISAs were clearly different between the melioidosis group and healthy donors. We observed that the difference in the median OD values was highest with OPS antigen, although the ROC analysis indicated comparable results among OPS, CF, and WC antigens. In addition, our results also indicated that, of the four antigen preparations tested, CPS was the least effective antigen for serodiagnosis of melioidosis. These findings are in agreement with our previous study, which evaluated the accuracy of ELISAs based on affinity-purified and crude B. pseudomallei antigen preparations (25).

IgG antibody has been shown to be a better indicator of active disease than IgM antibody for the diagnosis of melioidosis (38–40). IgM levels have been found to be higher than IgG levels in the healthy control serum (38). Therefore, in this study, we assayed for only IgG in melioidosis patient samples, other bacterial infection patient samples, and healthy controls to evaluate the ELISAs. Compared with standard bacterial culture and biochemical identification, our ELISAs using precoated plates reduced the time for laboratory diagnosis to 1.5 h, which is significantly less than the assay time for IHA (3.5 h).

Consistent with previous reports, our results suggest that IHA is not an ideal serological assay for use in laboratories in areas where melioidosis is endemic, such as in Thailand, although it might be useful in regions where the disease is nonendemic (3, 24). Using the same CF antigen preparation, comparison of AUROCC values for the CF-ELISA and IHA in Thailand revealed that the CF-ELISA performed better than the IHA. It is possible that adsorption of the CF antigens onto sheep red blood cells and/or the IHA method itself may contribute to the different results obtained for these two assays. IHA measured all immunoglobulins, while our ELISAs determined only IgG. Correlation analysis also demonstrated that the antibodies detected by IHA correlated only moderately with the results from all of the ELISAs using different crude antigens and purified polysaccharides.

Our previous study using an OPS-based latex agglutination assay with Thai melioidosis patients showed 84.4% sensitivity. While this assay was highly specific when tested with serum samples from individuals in areas where melioidosis is nonendemic (98%), the specificity was only 57% when tested with healthy Thai donor serum samples (24). With the different ELISAs developed in this study, the ROC analyses indicated that, when using purified OPS antigen, the specificity can be increased to 85.1% while maintaining a sensitivity of 81.6% with Thai donors. When the specificity was increased to 95.7%, the sensitivity of the OPS-ELISA was 71.6% for Thai donors, which is an improvement over the IHA. In addition, we demonstrated that OPS was the predominant B. pseudomallei antigen recognized by antibodies in melioidosis patient serum. Using the WC-ELISA with an OPS mutant preparation, we did not detect titers in two patients and found significantly lower titers in three of five patients. The specificity of the OPS-ELISA was not reduced when tested with serum samples from patients with other bacterial infections. Taken together, these findings suggest that the OPS-ELISA is a promising method that might be useful as a serodiagnostic tool in areas where melioidosis is endemic.

In contrast to the OPS antigen, the purified CPS antigen performed poorly for serodiagnosis of melioidosis. In fact, the sensitivity of the CPS-ELISA was lower than that of all of the other ELISAs tested in this study. The reason for this is unclear; however, it is possible that the levels of CPS-specific antibodies in patient serum samples may be lower than the antibody levels to other antigens (i.e., OPS, WC, CF). Simply increasing the serum concentration for the assay did not improve the sensitivity of the CPS-ELISA; instead it increased false-positive results from healthy donor serum samples (data not shown). Correlation analysis demonstrated that the antibody to CPS was only moderately correlated with the antibodies against the other antigens tested. Interestingly, the performance of CPS-ELISA was also poorer than that obtained in our previous study using a CPS-based latex agglutination test (24). This is likely attributable to the fact that the CPS-based latex agglutination test detected specific IgM and IgG, but the CPS-ELISA measured only IgG. Additional studies will be necessary to address this issue.

Our data indicated that one-fifth of on-admission melioidosis patients had negative specific IgG for all ELISAs. It is unclear why these patients did not have antibody responses. It is possible that the reliability of serology tests in immunocompromised patients might be low or that the serum samples were obtained too early in the infection process for detectable specific antibody. These potential explanations warrant further investigation. Analysis of the combined results of all ELISAs did not give a better result than using only the OPS-ELISA. Collectively, the results presented herein suggest that OPS is an appealing candidate for further development of a rapid point-of-care test. The benefits of using OPS as a target antigen include the following: it is very stable in comparison to protein preparations, it is expressed by only a limited number of pathogenic Burkholderia species (i.e., B. pseudomallei), and it can be purified from nonpathogenic sources (i.e., Burkholderia thailandensis). It should be noted that there is OPS antigen variations among B. pseudomallei isolates in different geographic regions. For example, different proportions of serologically distinct LPS serotypes (type A, B, B2, or rough) might result in more false-negative results in Australia (85.3% of isolates express type A and 13.8% express type B) than in Thailand (97.7% of isolates express type A) (41). Development of assays based on type B OPS antigen may be considered for certain areas. Alternative antigens that might be useful for serodiagnosis of melioidosis are CF and WC antigens, but these preparations have the disadvantages of being more difficult to standardize. A prospective evaluation using these antigens in ELISAs will be necessary to determine which antigen(s) is the most effective in various geographic regions.

The limitations of ELISA are recognized, including that it requires serum dilution steps and a microtiter plate reader. In addition, OD cutoff values may need to be determined for different areas depending on the exposure of local populations to B. pseudomallei and/or antigenically related species. However, ELISA is a standard serological assay, which may be the basis for further development of a rapid point-of-care test such as a lateral flow assay.

## Supplementary Material

Supplemental material
