# Supplementary material for: Development of Rapid Enzyme-Linked Immunosorbent Assays for Detection of Antibodies to Burkholderia pseudomallei
Source: J Clin Microbiol. 2016 Apr 25;54(5):1259–68. doi: 10.1128/JCM.02856-15 (PMC4844749; doi:10.1128/JCM.02856-15)
Supplement: Supplemental material [file supp_54_5_1259__index.html]

Supplemental material 

# Development of Rapid Enzyme-Linked Immunosorbent Assays for Detection of Antibodies to Burkholderia pseudomallei

## Supplemental material

- Supplemental file 1 -

  Fig. S1 (Correlation between results of ELISAs based on four different antigens and IHA titers, using 419 serum samples from melioidosis patients, Thai healthy donors, and U.S. healthy donors) and S2 (Results of ELISAs of four different antigens, using 539 sera from melioidosis patients, Thai healthy donors, U.S. healthy donors, tuberculosis patients, scrub typhus patients, and leptospirosis patients)

  PDF, 531K
