# Supplementary material for: Development of Rapid Enzyme-Linked Immunosorbent Assays for Detection of Antibodies to Burkholderia pseudomallei
Source: J Clin Microbiol. 2016 Apr 25;54(5):1259–68. doi: 10.1128/JCM.02856-15 (PMC4844749; doi:10.1128/JCM.02856-15)
Supplement: Supplemental material [file JCM.02856-15_zjm999094913so1.pdf]

Figure S1 Correlation between the results of ELISAs based on four different antigens and IHA titers using 419 serum samples from melioidosis patients, Thai healthy donors and U.S. healthy donors. Box plots represent 25th and 75th percentile boundaries in the box with the median line within the box; the whiskers indicate the 10th and 90th percentiles. The plots show OD 450 nm for each antigen: (A) OPS, (B) CPS, (C) WC, (D) CF antigens and different IHA titers.

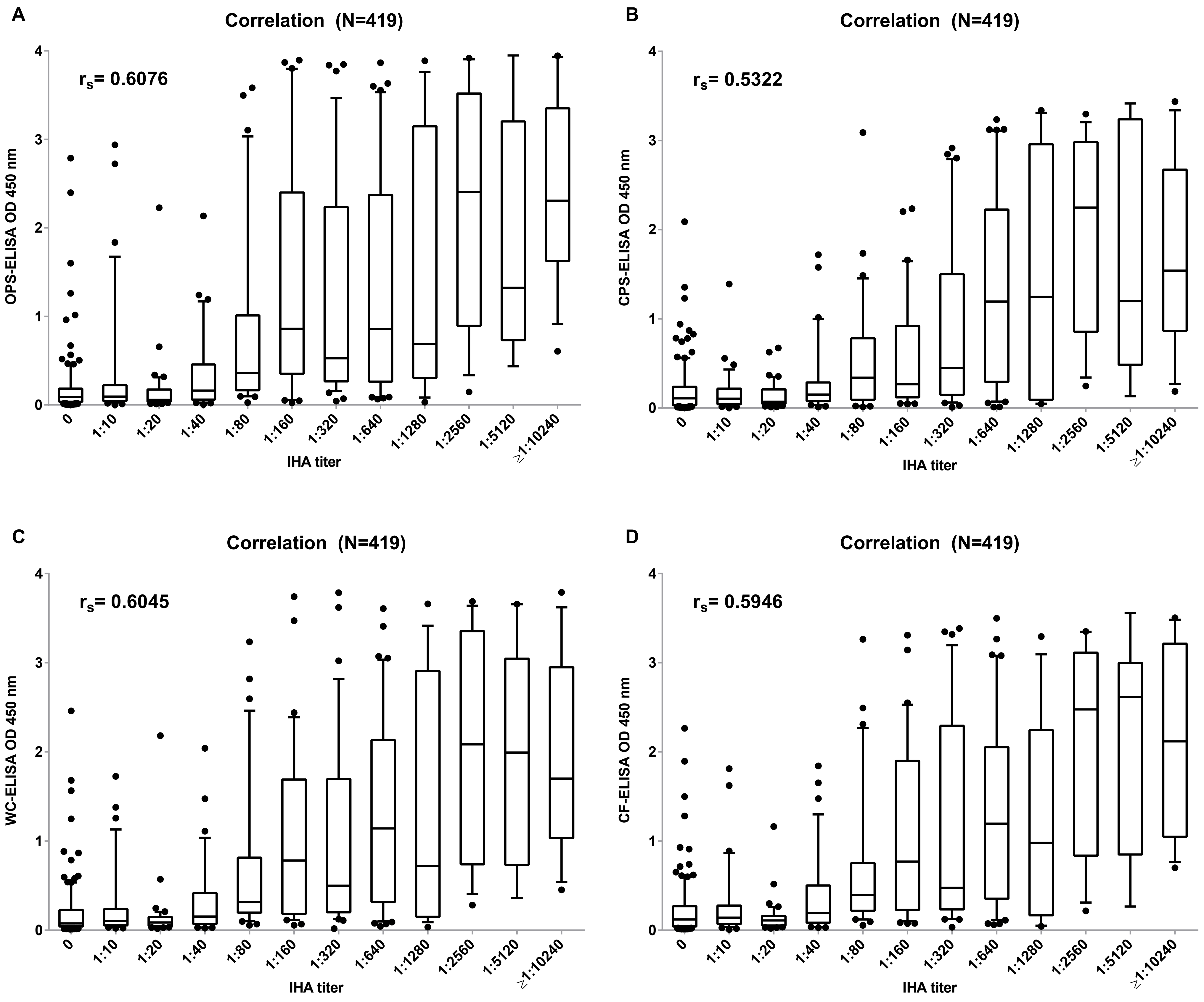

A
Correlation (N=419)
r_s= 0.6076
OPS-ELISA OD 450 nm
IHA titer
B
Correlation (N=419)
r_s= 0.5322
CPS-ELISA OD 450 nm
IHA titer
C
Correlation (N=419)
r_s= 0.6045
WC-ELISA OD 450 nm
IHA titer
D
Correlation (N=419)
r_s= 0.5946
CF-ELISA OD 450 nm
IHA titer
0
1:10
1:20
1:40
1:80
1:160
1:320
1:640
1:1280
1:2560
1:5120
≥1:10240

Figure S2 Results of ELISAs of four different antigens using 539 sera from melioidosis patients, Thai healthy donors, U.S. healthy donors, tuberculosis patients, scrub typhus patients and leptospirosis patients. Orange boxes represent positive and white boxes represent negative OD values of individual serum samples. The cut-off OD was set at 3 different values at 85%, 90% and 95% specificity using the Thai healthy donor group as a control.

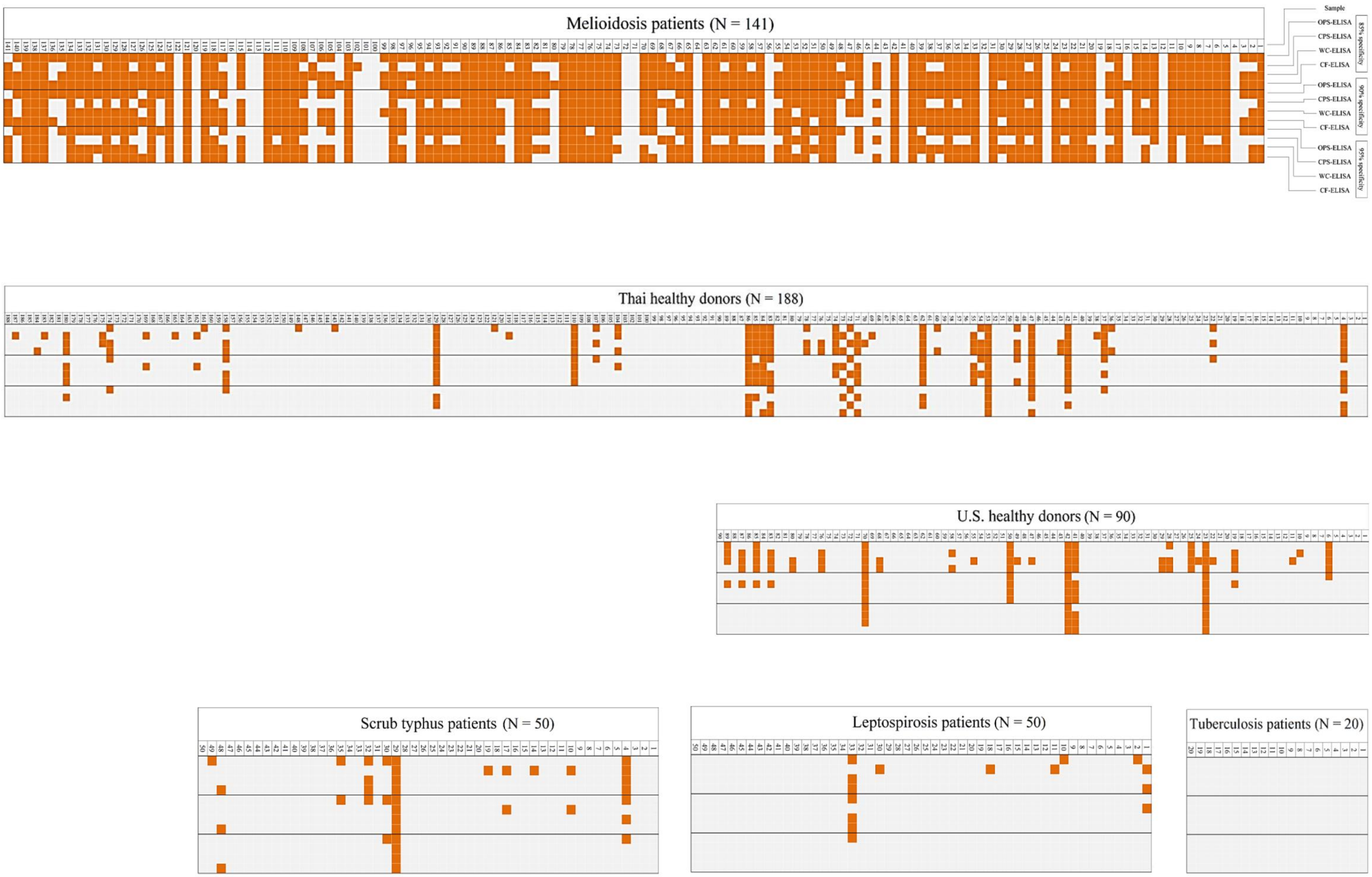
Melioidosis patients (N = 141)
Thai healthy donors (N = 188)
U.S. healthy donors (N = 90)
Scrub typhus patients (N = 50)
Leptospirosis patients (N = 50)
Tuberculosis patients (N = 20)
Sample
85% specificity
OPS-ELISA
CPS-ELISA
WC-ELISA
CF-ELISA
90% specificity
OPS-ELISA
CPS-ELISA
WC-ELISA
CF-ELISA
95% specificity
OPS-ELISA
CPS-ELISA
WC-ELISA
CF-ELISA
